# Effects of Nitrogen Supplementation Status on CO_2_ Biofixation and Biofuel Production of the Promising Microalga *Chlorella* sp. ABC-001

**DOI:** 10.4014/jmb.2005.05039

**Published:** 2020-06-15

**Authors:** Jun Muk Cho, You-Kwan Oh, Won-Kun Park, Yong Keun Chang

**Affiliations:** 1 Department of Chemical and Biomolecular Engineering, Korea Advanced Institute of Science and Technology (KAIST), Daejeon 34141, Republic of Korea; 2 School of Chemical and Biomolecular Engineering, Pusan National University, Busan 46241, Republic of Korea; 3 Department of Chemistry and Energy Engineering, Sangmyung University, Seoul 03016, Republic of Korea; 4 Advanced Biomass R&D Center, Daejeon 34141, Republic of Korea

**Keywords:** *Chlorella*, nitrate, CO_2_ biofixation, biofuel, lipid, carbohydrate

## Abstract

The use of microalgal biomass as feedstock for biofuels has been discussed for decades as it provides a sustainable approach to producing fuels for the future. Nonetheless, its feasibility has not been established yet and various aspects of biomass applications such as CO_2_ biofixation should also be explored. Therefore, in this study, the CO_2_ biofixation and lipid/carbohydrate production potential of *Chlorella* sp. ABC-001 were examined under various nitrogen concentrations. The highest biomass productivity and CO_2_ biofixation rate of 0.422 g/l/d and 0.683 g/l/d, respectively, were achieved under a nitrogen-rich condition (15 mM nitrate). Carbohydrate content was generally proportional to initial nitrate concentration and showed the highest value of 41.5% with 15 mM. However, lipid content showed an inverse relationship with nitrogen supplementation and showed the highest value of 47.4% with 2.5 mM. In consideration as feedstock for biofuels (bioethanol, biodiesel, and biogas), the sum of carbohydrate and lipid contents were examined and the highest value of 79.6% was achieved under low nitrogen condition (2.5 mM). For lipid-based biofuel production, low nitrogen supplementation should be pursued. However, considering the lower feasibility of biodiesel, pursuing CO_2_ biofixation and the production of carbohydrate-based fuels under nitrogen- rich condition might be more rational. Thus, nitrogen status as a cultivation strategy must be optimized according to the objective, and this was confirmed with the promising alga *Chlorella* sp. ABC-001.

## Introduction

Fixation of carbon dioxide has become one of the most crucial technologies required by modern society and ological fixation by photosynthesis is the ideal form to reduce carbon dioxide over the long term [1]. Moreover, the only biological process that can effectively remove CO_2_ is through the cultivation of microalgae, as the use of first- and second-generation biomass suffers from critical drawbacks [2]. These tiny organisms exhibit higher photosynthetic efficiency, growth and lipid productivities compared to terrestrial plants that have been exploited for biofuels [3]. As microalgae utilize carbon dioxide for growth, the production of biofuels using algae will help reduce the accumulation of carbon dioxide in the atmosphere. Microalgae biomass is the most promising feedstock for biofuel that can help maintain a sustainable society.

Under optimized conditions, microalgae can accumulate significant amounts of lipids and carbohydrates, which can be converted to fuels. Lipids from microalgae are considered to be an excellent feedstock for biodiesel and have been discussed in several studies [4-6]. Biodiesel from microalgae is compatible with current diesel engines and thus can be implemented right away [7]. However, the economic feasibility of biodiesel has not been established yet and further efforts to improve the costs and productivity of generating these environmentally friendly fuels are necessary [4, 8].

There have been various efforts to broaden the application of microalgal biomass for the production of biofuels. Previous studies have discussed the application of microalgal biomass as combustion fuel in power plants [9, 10]. These studies suggest that biomass with high lipid is favorable as it has higher heating value and results in high energy yield. Besides lipids, carbohydrates also constitute a significant proportion of microalgae and can be employed to produce bioethanol by fermentation [11]. Furthermore, both lipid and carbohydrate can be converted to biofuels if carbohydrates are utilized after oil extraction [12]. Microalgal biomass as a whole or in part can also be used as an organic source in anaerobic digestion for biogas production [13]. In this case, high C:N ratio is preferred, which is commonly shown in biomass with high lipid and carbohydrate contents. As described, there are various methods to utilize microalgal biomass for the production of biofuels. However, high content of lipid and carbohydrates is important as they allow maximum utilization of biomass, which can lead to improved economic feasibility. Thus, it is crucial to utilize microalgae that have the potential to achieve such compositions of biomass.

The simplest and most effective method to achieve high lipid content in microalgal cells has been to cause nitrogen starvation by limiting the nitrogen input into the cultivation medium [14]. This led to increased lipid composition but decreased biomass production [15]. Hence, both nitrogen-replete and -deplete conditions were employed in two-stage cultivation systems to achieve maximum lipid productivity by separating the growth and lipid accumulating phases [16, 17]. However, changing the nitrogen availability of the medium on a large scale is critical to the cost of mass production. Therefore, nitrogen starvation in cells should be naturally induced by causing nitrogen depletion in the medium by cellular consumption. This would impact biomass productivity, which in turn affects lipid productivity and CO_2_ biofixation rate as a consequence. The trade-off between high biomass productivity and CO_2_ biofixation rate versus high lipid content has highlighted the importance of understanding the extent of effects caused by varying the nitrogen supply to cells.

This study focuses on the supply of nitrogen and its effects on the cell composition, lipid and carbohydrate productivity as well as the CO_2_ biofixation rate of *Chlorella* sp. ABC-001. The microalga *Chlorella* sp. ABC-001 is a newly isolated strain with advantageous characteristics for CO_2_ fixation and biofuel production. During cultivation, it showed various performance levels of growth, lipid and carbohydrate accumulation under different amounts of nitrogen supplementation. This controllable variation was used to discuss effective cultivation methods for application in biofuel production and CO_2_ biofixation.

## Materials and Methods

### Microalgae Strain and Cultivation Conditions

The microalgal strain used in this study was the green alga *Chlorella* sp. ABC-001, which was isolated from a stream near a power plant in Gangwon Province, South Korea. Modified N-8 medium was used to grow the cells and contained the following compounds in g/l: 0.74 KH_2_PO_4_, 0.2598 Na_2_HPO_4_, 0.05 MgSO_4_•7H_2_O, 0.0175 CaCl_2_•2H_2_O, 0.0115 FeNaEDTA•3Hs_2_O, 0.0032 ZnSO_4_•7H_2_O, 0.013 MnCl_2_•4H_2_O, 0.0183 CuSO_4_•5H_2_O, and 0.007 Al_2_(SO_4_)_3_•18H_2_O. In addition, potassium nitrate was added to create initial nitrate concentrations of 2.5, 5, 10 and 15 mM. Cells were cultivated in bubble-column photobioreactors (PBRs) with a working volume of 500 ml, which were autoclaved prior to cultivation. Light was provided from white fluorescent lamps at an intensity of 170 μmol photons/m^2^/s. Gas was supplied from the bottom of the PBRs for mixing and supply of carbon dioxide. The supplied gas was composed of 10% CO_2_ and balanced with air. Cultivations were carried out in a temperature- controlled room maintained at 30oC. The evaporation rate of water was monitored daily and the values were used to correct the following growth curve from concentration by water loss. All cultivation experiments were carried out in duplicates.

### Measurement of Cell Growth and Nitrate Concentrations

Cell growth was measured in terms of dry cell weight (DCW) which was monitored daily. To calculate the DCW, 3-5 ml of culture samples were filtered using pre-dried and weighed GF/C filters (Whatman, UK) and washed several times with deionized water. The filtered biomass was dried overnight in a 70oC oven and weighed. The difference in the weights of the filters was used to calculate the DCW.

For measurement of nitrate concentrations, 5-10 ml of cultures were centrifuged and the supernatant was filtered using 0.2 μm filters (Minisart NML, Germany). The filtered supernatant was diluted accordingly using deionized water and analyzed using ion chromatography (883 Basic IC Plus, Metrohm, Switzerland) with an anion column (Metrosep A Supp 5, Switzerland). An eluent solution with a composition of 3.2 mM Na_2_CO_3_ and 1 mM of NaHCO_3_ was fed into the column at a flow rate of 0.7 ml/min.

### Elemental Analysis of Biomass

Biomass was harvested by centrifugation on day 7 and 14 and washed twice with distilled water to remove any residual nutrients from the medium. Then, the cells were lyophilized using a freeze-dryer for 3 days and analyzed using an elemental analyzer (FLASH 2000 Series, Thermo Scientific, USA) to determine the compositions of C and N in the biomass. Each sample was analyzed twice and the average value was used to represent the data value for each sample.

### Biomass Productivity and CO_2_ Biofixation Rate

Biomass productivity, Pb (g/l/d), was calculated using the following equation:

(1) $$P_{b}=\frac{X_{1}-X_{o}}{t_{1}-t_{o}}$$

X1: biomass (g/l) at time t1, Xo: initial biomass at time to (t = 0).

The carbon dioxide biofixation rate (g/l/d) can be calculated with the following equation:

(2) $$R_{CO_{2}}=C_{carbon}\times P_{b}\times \left(\frac{m_{CO_{2}}}{m_{C}}\right)$$

C_carbon_: carbon content of biomass, Pb: biomass productivity (g/l/d), Mc and MCO_2_ : molecular weights of carbon and CO_2_.

### Analysis of Lipid and Carbohydrate

Biomass was harvested on day 5, 7, 10 and 14 for analysis of total lipid and carbohydrate contents. Harvested biomass was washed with distilled water and lyophilized for 3 days. The dried biomass was finely ground into powder and stored at −70oC in a deep freezer until analysis was carried out. The determination of total lipid content was done with a modified Folch’s method. In detail, 50 mg of the dried biomass was added with 10 ml of chloroform/methanol mixture into a Pyrex glass tube and sonicated for 4 h in a bath sonicator. After the addition of 2.5 ml of distilled water, the sample was mixed vigorously and centrifuged for phase separation. The lower organic phase which consisted of chloroform and extracted lipid, was transferred to a fresh glass tube after passing through 0.2 μm organic solvent filters (Minisart RC, Germany). Then, 5 ml of the filtered solvent was transferred into a pre-weighed tube and sparged with a steady flow of nitrogen gas until all of the chloroform was evaporated. Finally, the dried lipid was weighed and the lipid content was calculated according to the following equation:

(3) $$\text{Total lipid content (\%)}=\frac{\left(W_{L}-W_{D}\right)\times V_{C}}{V_{L}\times W_{s}}\times 100(\%)$$

W_D_: the weight of empty tubes, W_L_: the weight of tubes with extracted lipid, V_C_: volume of chloroform, V_L_: volume of chloroform and lipid transferred to tubes, Ws: the weight of biomass sample.

W_D_: the weight of empty tubes, W_L_: the weight of tubes with extracted lipid, V_C_: volume of chloroform, V_L_: volume of chloroform and lipid transferred to tubes, Ws: the weight of biomass sample.

The total carbohydrate content was determined using the phenol-sulphuric acid method. Approximately 3-5 mg of dried biomass powder was weighed and mixed vigorously in 10 ml distilled water after which 1 mL was transferred to fresh glass tubes. Subsequently, 1 ml of phenol solution (5% wt.) and 5 ml of concentrated sulphuric acid was added and left in the dark to react for 30 min. The solution was mixed well by inverting the tubes several times and the absorbance at 470 nm was measured using a UV/Vis spectrophotometer (Shimadzu, Japan). Five standards were also prepared using glucose solution and a standard curve was produced as the following equation:

(4) $$\text{y = 0}{\text{.162x (R}}_{2}\text{ = 0.991)}$$

y: carbohydrate concentration (g/l), x: absorbance at 470 nm.

The carbohydrate content was derived by dividing the calculated carbohydrate concentration by the biomass concentration in the sample.

## Results and Discussion

### Cultivation of *Chlorella* sp. ABC-001 Under Various Nitrate Concentrations

**Fig. 1:**
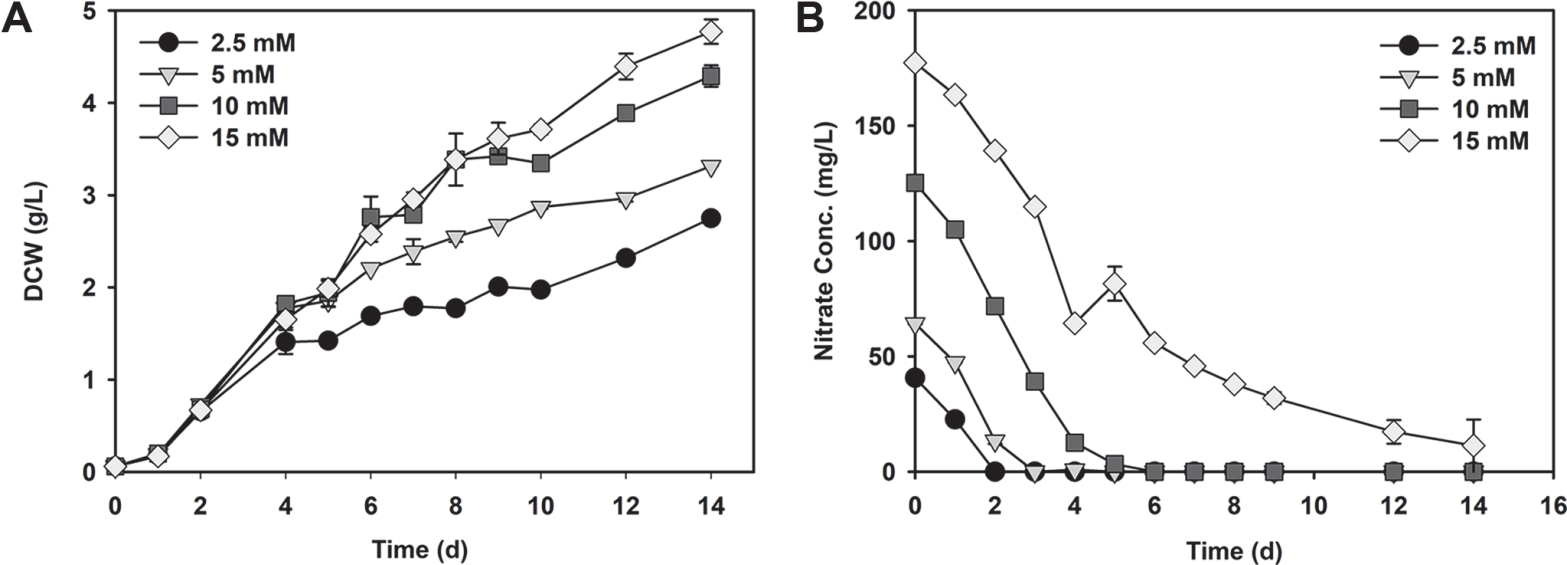
Growth profile of *Chlorella* sp. ABC-001 cells grown under 2.5 to 15 mM nitrate concentrations. (**A**) Cell growth and (**B**) nitrate consumption.

The high CO_2_ tolerant *Chlorella* sp. ABC-001 cells were cultivated for 14 days to study the changes in biomass production and CO_2_ biofixation as well as cell composition with various nitrate conditions. The growth of *Chlorella* sp. ABC-001 cells in terms of DCW is shown in Fig. 1A. The initial concentrations of nitrate in the medium were varied between 2.5 to 15 mM and the effects on biomass generation were observed. The maximum final biomass concentration of 4.77 g/l was achieved under 15 mM of nitrate whereas the lowest final biomass concentration of 2.75 g/l was obtained with 2.5 mM after 14 days. Over the course of the cultivation period, continuous growth was achieved under all experimental conditions. However, the increase in DCW between various nitrate concentrations started to differ from day 2 and the final DCW was increased when the initial nitrate concentration was higher. Nitrogen starvation leads to decreased synthesis of chlorophyll and negatively affects the photosynthetic system in microalgae [18]. The optimal conditions for growth are generally considered with the C:N:P ratio of cells but in this case, carbon was continuously supplied in the form of CO_2_, and thus the N:P ratio was the determining factor. The phosphorus concentration in the medium was 7.31 mM, which showed the N:P ratio to be between 0.34-2.05. This was much lower than the general values for N:P ratio between 5-50 [19] and thus, it could be said that phosphorus in the medium was abundant for cell growth considering the concentration of nitrogen in the medium. Therefore, nitrogen was the only limiting nutrient and consequently determined the growth of cells. As expected, increasing the availability of nitrogen from 2.5 to 15 mM resulted in higher biomass accumulation (2.75 to 4.77 g/l on day 14).

The consumption of nitrate over time was also observed daily by measuring the remaining nitrate in the supernatant (Fig. 1B). All of the added nitrates were consumed by the cells at the end of the cultivation period except for the addition of 15 mM nitrate, where the final residual nitrate concentration was 11.3 mg/l. However, even in this case, the nitrate concentration approached zero and it appears that all nitrogen would be assimilated into the cells if more time was given. Depletion of nitrate in the medium for 2.5, 5 and 10 mM occurred on day 2, 3 and 6 of cultivation, respectively. Considering that cells grew well under the various nitrate concentrations, this result suggests that other conditions such as light, temperature, carbon dioxide supply were sufficient for this species and the sole effect of nitrogen limitation should be studied in detail. The initial rate of consumption was similar (14-20 mg/l/d) in all cases and with increasing initial nitrate concentrations the time of depletion increased as well. To maximize the production of biomass, it is shown here that increasing nitrate concentration is very effective. For all cases, nitrate was consumed well by the cells and was the sole key factor that determined the final biomass yield as other conditions were fulfilled. These results corresponded with the previous study in which the authors showed that fed-batch feeding of nitrate resulted in an increased growth rate which led to a 56% increase in carbon dioxide fixation rate [20].

### Elemental Composition of *Chlorella* sp. ABC-001 and CO_2_ Biofixation

In the past, the primary interest in microalgae was to utilize the biomass for producing biodiesel from cellular lipids. However, with low oil prices and increasing concerns over global warming, the focus has shifted to the biological fixation of CO_2_. As the fixation of CO_2_ by algal cells is dependent on their carbon content and biomass productivities, the elemental composition of carbon was determined for various nitrate concentrations. Elemental analysis of cells grown for 7 and 14 days was carried out to determine the internal carbon and nitrogen content (Table 1). The carbon content of *Chlorella* sp. ABC-001 was inversely proportional to the initial concentration of nitrate in the medium and carbon content increased as nitrate in the medium became depleted. Only the lowest nitrate concentration (2.5 mM) conveyed more than 50% at day 7, but on day 14, all of the nitrate-depleted conditions (2.5, 5, and 10 mM) showed more than 50% carbon content (57.6%, 54.5%, and 51.9%, respectively) with continuous increase in carbon content over time. This can be explained by the fact that when cells are faced with nitrogen-limited condition, they uptake carbon and store it in the form of lipid and carbohydrate [6]. Hence, cells with high carbon content are likely to contain a high proportion of lipid which is confirmed in the following section. In addition, the nitrogen content of cells decreased with the initial concentration of nitrate and time as available nitrogen was depleted. Due to the fact that the only source of nitrogen is the nitrate in the medium and cells continue to assimilate carbon even after nitrogen depletion, the nitrogen content, a relative value, is decreased over time. This resulted in increased C/N ratios, especially in low nitrogen conditions. The minimum and maximum values of C/N ratio were 8.87 with 15 mM at day 7 and 31.1 at 2.5 mM at day 14, respectively.

To calculate the CO_2_ biofixation rate of cells, average biomass productivities at day 7 and 14 were calculated according to Eq. (1). The resulting values along with the carbon content of cells were substituted into Eq. (2) to obtain the carbon dioxide fixation rates at day 7 and 14 (Table 2). As biomass productivities varied significantly between nitrate concentrations (0.196 to 0.422 g/l/d), the CO_2_ biofixation rate followed a similar trend (0.415 to 0.683 g/l/d). Increasing nitrogen availability from 2.5 to 15 mM positively affected the fixation rate (34-49% increase) while prolonged cultivation reduced the rate (9-24% decrease). The highest CO_2_ biofixation rate was observed on day 7 under 15 mM of nitrate, but on day 14 the fixation rate was reduced by 9.3% to 0.619 g/l/d from 0.683 g/l/d. These results also corresponded with previous research [20]. This indicated that for maximum CO_2_ removal rate, a short cultivation batch with high concentration of nitrate is most effective. However, when considering the production of biofuels from microalgae as well, this may not be the case as nitrate concentration affects cell composition, which is discussed in detail in the following sections.

### Lipid Content and Productivity

**Fig. 2:**
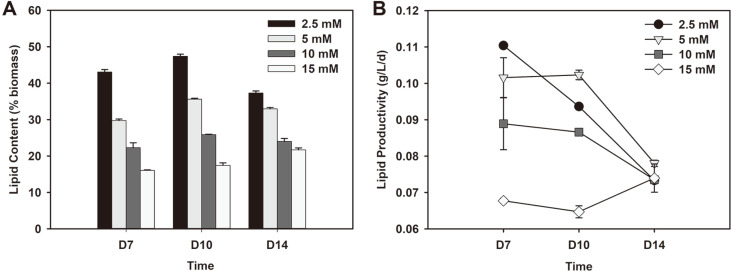
Total (A) lipid content and (B) lipid productivity on day 7, 10 and 14 of *Chlorella* sp. ABC-001 grown under 2.5 to 15 mM nitrate concentrations.

Lipid from microalgae can be used as feedstock for biofuel and the availability of nitrogen plays a crucial role in lipid accumulation in microalgae [6]. Hence, the effects of nitrate concentration on lipid production by *Chlorella* sp. ABC-001 cells were examined. Fig. 2A shows that the lipid content varies significantly between nitrate concentrations but mostly follows a general trend in which nitrogen-limited conditions are essential for high lipid biomass. At higher concentrations of nitrate, the maximum lipid contents (21.7-35.6%) achieved were significantly less than cells grown with 2.5 mM nitrate (47.4%). Cells also require a period of time after depletion of nitrogen to alter their compositions to become lipid oriented as they utilize internal nitrogen pools after depletion of nitrogen in their surrounding environment [21]. Therefore, to achieve high lipid content, depletion of nitrogen in the medium as well as sufficient exposure time for lipid accumulation are both required. Prolonged exposure to nitrogen-depleted environments led to increasing lipid compositions except on day 14 when decreases were observed in cases of lower initial nitrate additions (2.5, 5 mM). This may be due to cell deterioration caused by prolonged stress experienced by cells in a nitrogen-depleted condition [18]. When nitrogen is depleted, it has been shown that cells stop dividing and accumulate lipid or carbohydrate, which leads to increased cell sizes [22].

However, from a productivity perspective, limiting the nitrogen will lead to reduced biomass production and negatively affect the lipid productivity. Hence, to optimize the overall productivity of lipid, the lipid content as well as biomass productivity should be considered at the same time. As shown in Fig. 2B, lipid productivities tended to increase as lipid content increased after nitrogen depletion. However, over time, lipid productivities decreased as did biomass productivities and lipid content. The highest lipid productivity of 0.110 g/l/d was observed with 2.5 mM on day 7. On the final day, lipid productivities under all conditions reached a similar value of 0.07-0.08 g/l/d as a result of decreasing biomass productivities over time. These values are much higher than 0.056 g/l/d observed with *Chlorella* sp. ESP-31 [23] or comparable to 0.118 g/l/d with *Chlorella* sp. MRA-1 [24].

**Fig. 3:**
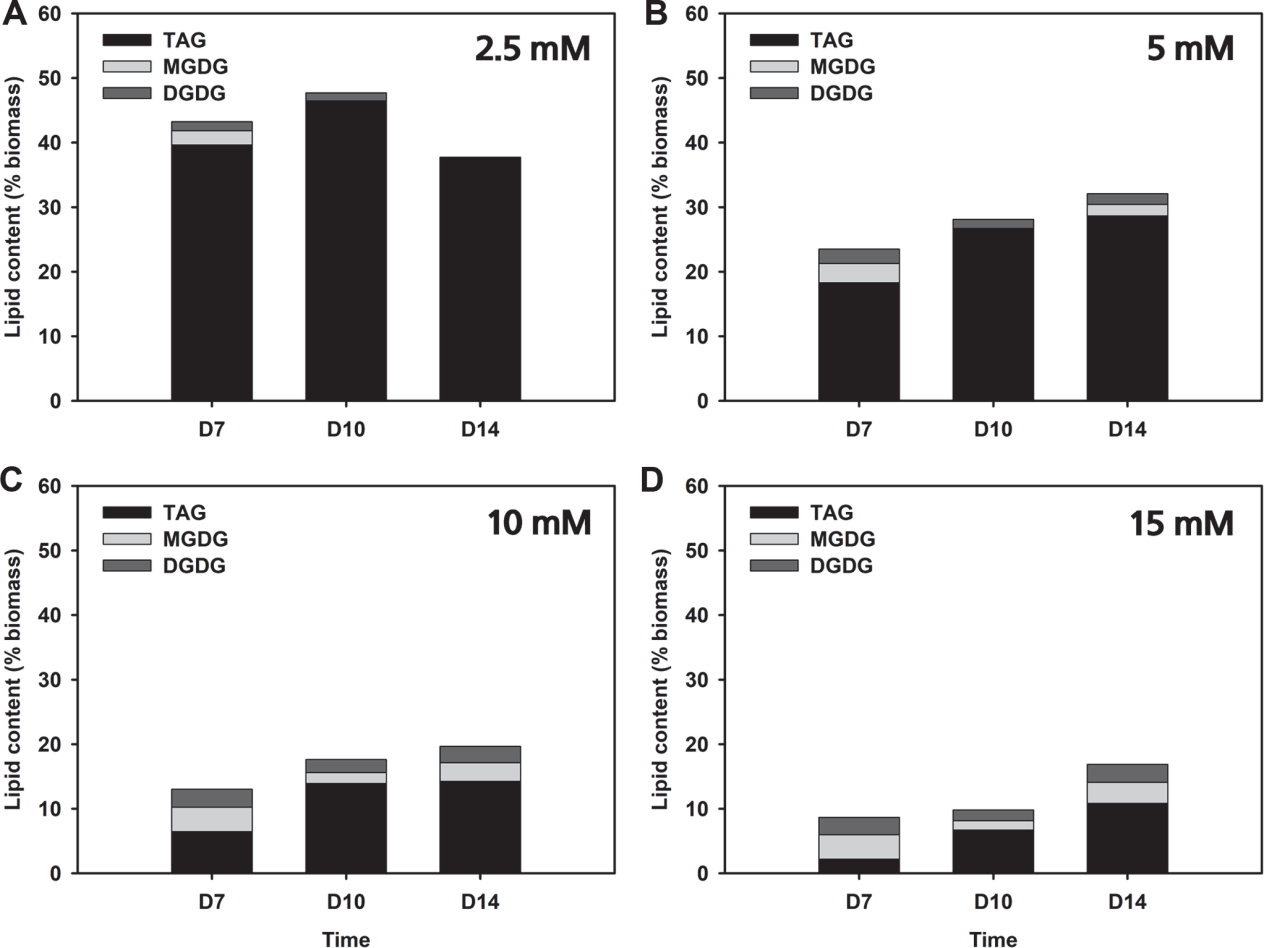
Lipid composition of *Chlorella* sp. ABC-001 biomass harvested on day 7, 10 and 14 with (A) 2.5 mM, (B) 5 mM , (C) 10 mM, and (D) 15 mM initial nitrate supplementation.

Also, lipids found in microalgae can be classified into various types including neutral lipids, polar lipids and pigments [25]. Among them, neutral lipids that consist of long-chain fatty acids are the preferred type for biofuel as they generate high biofuel yield [26]. Additionally, neutral lipids do not contain high concentrations of phosphorus which can be toxic to catalysts used to convert microalgal lipids to fuels [27]. As shown in Fig. 3, three types of lipids were classified within the *Chlorella* sp. ABC-001 biomass: triacylglycerol (TAG), monogalactosyl- diacylglycerol (MGDG) and digalactosyldiacylglycerol (DGDG). TAG is a storage lipid and the major component of lipid found in microalgae, whereas MGDG and DGDG are membrane lipids found in chloroplasts [28]. TAG is also the major component of lipid in *Chlorella* sp. ABC-001, cultivated in this study, especially under low nitrate concentrations. Increases in TAG content over time can be seen in all cases except on day 14 with 2.5 mM, which may be caused by cell deterioration due to prolonged exposure to nitrogen deficiency. Moreover, the proportion of TAG in total lipid from cells grown under limited nitrogen (61-100% with 2.5 and 5 mM nitrate) was much greater than in cells grown in the abundance of nitrogen (14-60% with 5 and 15 mM nitrate). Furthermore, the ratio of TAG to total lipids is much higher than reported in literature [25, 29]. Hence, the increase in total lipid content can be solely attributed to the increase in TAG content of cells. MGDG and DGDG were found in larger concentrations in the biomass grown with greater nitrate concentration. At higher concentrations of nitrate, the sum of concentrations of MGDG and DGDG were maintained above 6% of biomass even after 14 days as shown in 10 and 15 mM nitrate conditions, while the highest sum of these membrane lipids was 6.3% of biomass achieved on day 7 with 15 mM nitrate. However, with a low concentration of nitrate, they seemed to decrease over time as nitrogen was depleted. On day 14 with 2.5 mM of initial nitrate addition, the sum of MGDG and DGDG was only 0.15% of the total biomass, which was the lowest value observed. Therefore, the availability of nitrogen affected the concentration of both storage and membrane lipids but in an opposite manner. These results are consistent with previous findings [30, 31] suggesting that the glycolipids are converted into TAGs when cells are nitrogen deprived.

### Carbohydrate Content and Productivity

**Fig. 4:**
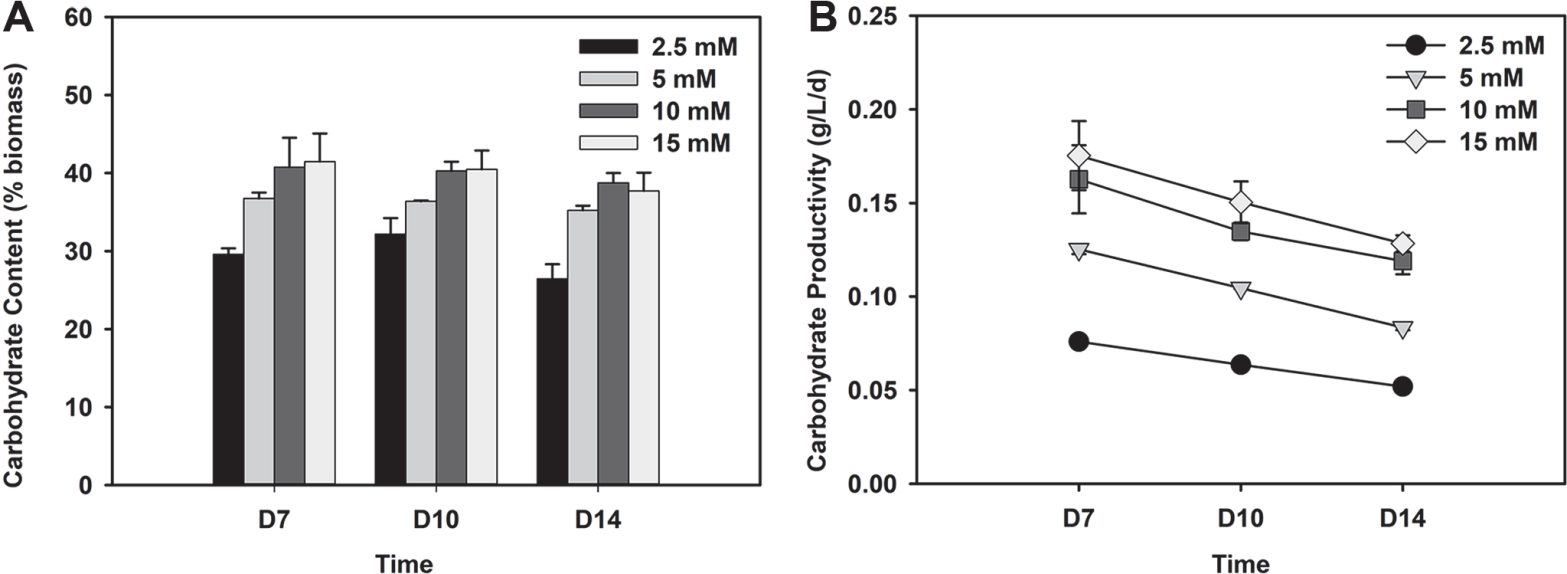
Total (A) carbohydrate content and (B) carbohydrate productivity on day 7, 10 and 14 of *Chlorella* sp. ABC-001 grown under 2.5 to 15 mM nitrate concentrations.

While lipids are considered to be the major component of biofuel feedstock in microalgal cells, carbohydrates also constitute a significant proportion of cells [32]. These carbohydrates can be used for the production of bioethanol, biohydrogen via fermentation or biochar via thermochemical conversion [12, 33]. *Chlorella* sp. ABC- 001 cells also exhibit high content and productivity of carbohydrates and the changes under various nitrate concentrations were studied. Carbohydrate content showed the opposite trend from lipid content as the composition of carbohydrate decreased with lower nitrogen conditions (Fig. 4A). The changes in carbohydrate content were not as dramatic as with lipid in most cases exhibiting total carbohydrate content between 30-40% of cell composition. The highest carbohydrate content (41.5%) was observed under 15 mM on day 7 and gradually decreased as cells were exposed to lower concentrations of nitrate. Thus, 2.5 mM of nitrate showed the lowest carbohydrate content of 26.5% on day 14. In addition, the carbohydrate content showed a decrease over time under low nitrate concentrations, specifically 2.5 mM. These phenomena may be attributed to the changes in cell metabolism to facilitate the increased accumulation of lipids [34]. As both biomass productivity and carbohydrate content were higher with greater nitrate supply, the total carbohydrate productivity was proportional to the amount of nitrate added (Fig. 4B). The highest carbohydrate productivity of 0.175 g/l/d was shown with 15 mM nitrate on day 7. This value is much greater than 0.091 g/l/d observed with *Neochloris oleoabundans* HK-129 [17] or 0.038 g/l/d with *Chlorella fusca* LEB111 [35]. However, productivity tended to show a steady decline over time under all cases as biomass productivity decreased. Hence, the lowest carbohydrate productivity was found with 2.5 mM nitrate on day 14. Furthermore, the lowest carbohydrate productivity observed with 15 mM nitrate (0.128 g/l/d) was still higher than the highest carbohydrate productivities with 2.5 and 5 mM nitrate (0.076 g/l/d and 0.125 g/l/d, respectively) on day 7. This proved that increasing nitrogen supply has a significant effect on the production of carbohydrate with *Chlorella* sp. ABC-001.

### Potential Applications of Microalgae: Carbon Biofixation and Biofuel Feedstock

Cellular composition of microalgae can be divided into lipids, carbohydrates and proteins, and each component has various uses. Nonetheless, for biofuel purposes, the compositions of both lipids and carbohydrates are considered. Lipids have mainly been used as a source for biodiesel. TAG, the main form of storage carbon, is extracted and trans-esterified into fatty acid methyl esters that can be used as diesel fuel directly. Carbohydrates could be used as a renewable source for bioethanol production or biogas by fermentation. To obtain biomass suitable as feedstock for biofuel, high content of lipid and carbohydrate is preferred and thus inducing nitrogen depletion early can initiate accumulation of the target compounds within cells. Biomass with both high lipid and carbohydrate content yields high energy return and maximizes the utilization of generated biomass, leading to reduced waste of biomass and improved economic feasibility as well. The *Chlorella* sp. ABC-001 biomass examined in this study exhibited significant compositions of lipid and carbohydrate, and thus potential application for biofuels was discussed with the sum of the two compounds. While each substance can be converted to different forms of biofuels such as biodiesel or bioethanol, some studies suggest utilizing the entire biomass by thermal conversion [36] or as combustion fuel [9]. In this case, to effectively utilize microalgal biomass as combustion fuel, it is important for cells to have high lipid and carbohydrate content to achieve high heating values [10]. Furthermore, if the biomass grown using flue gas is used as combustion fuel for power plants, it could potentially reduce the carbon footprint of the largest source of carbon dioxide emissions. Many studies have successfully achieved high content of lipid or carbohydrate by controlling the supply of nutrients but few have shown results where both lipid and carbohydrate constitute the major proportion of cells [3]. The sum of lipid and carbohydrate contents for *Chlorella* sp. ABC-001 was maintained above 60% in most cases with a maximum of 80% in cells grown with 2.5 mM for 10 days. Results showed that the sums decreased with increasing nitrate concentration due to low lipid content in the biomass cultivated with abundant nitrogen. The lowest sums were all observed with 15 mM nitrate where the lipid content was the lowest. Therefore, limiting the nitrogen concentration can be considered as a crucial factor when cultivating microalgae for biofuels.

These results were compared to previous studies that employed various strains of microalgae (Table 3). The two sets of results of *Chlorella* sp. ABC-001 attained in this study were representative of maximum CO_2_ biofixation rate (15 mM nitrate) and the maximum sum of lipid and carbohydrate content (2.5 mM nitrate). Many studies have focused on maximizing either lipid or carbohydrate for biofuel feedstock but not many have discussed the overall potential of microalgal biomass in terms of both lipid and carbohydrate. The results of this study showed that *Chlorella* sp. ABC-001 accumulated significant contents of both lipid and carbohydrate under 2.5 mM nitrate condition and the sum of these two cellular compounds was considerably higher than most other strains. Only *Dunaliela tertiolecta* showed a comparable sum of 78.9%, but was mainly carbohydrate oriented.

In addition to the composition of cells, the biomass productivity and CO_2_ biofixation rate should also be considered to improve the feasibility of microalgal biofuel production. The *Chlorella* sp. ABC-001 cells cultivated in this study showed the highest biomass productivity and CO_2_ biofixation rate among other strains except for *Scenedesmus vacuolatus*. However, despite considerable biomass generation, *S. vacuolatous* showed low lipid (28.04%) and carbohydrate content (20.28%), which decreased its applicability for biofuel production. As there is an obvious trade-off between high lipid content and biomass production, a suitable nitrogen concentration should be selected according to the target objective and microalgal strain. Thus, in this study, in a process where the removal of CO_2_ is the main focus, sufficient nitrate is preferred as it results in greater biomass production (65% increase) and CO_2_ fixation (34% increase) as shown by the values in Table 3, but the generation of lipid feedstock could be largely decreased (44% less). In this case, carbohydrates, which are enhanced under high nitrogen concentrations, may be chosen as the main target product. Based on the values for *Chlorella* sp. ABC-001 in Table 3, this would result in up to 112% more carbohydrate (0.0824 to 0.175 g/l/d) and 19% more combined lipid and carbohydrate productivity (0.204 to 0.243 g/l/d). Consequently, CO_2_ biofixation and simultaneous production of carbohydrate- and lipid-based fuels would be a much better option than solely focusing on lipid-based biofuel as the major product. In addition, with the current low fuel prices, prioritizing CO_2_ fixation performance may become more beneficial than the production of biofuels [37, 38].

As there are various methods to utilize the different biochemicals produced with microalgae, it is first and foremost important to understand how to maximize the production of target compounds under various environmental conditions. Subsequently, the feasibility of microalgal cultivation should be discussed on an industrial scale within the context of both biofuel generation and CO_2_ removal as they would benefit both the economical and environmental aspects. This study discusses in detail the effect of nitrogen supplementation status on the cell composition, productivity, and CO_2_ biofixation of *Chlorella* sp. ABC-001, thus providing crucial and fundamental data for optimizing the strategy for biofuel production and CO_2_ fixation with microalgae.

## Figures and Tables

**Fig. 5:**
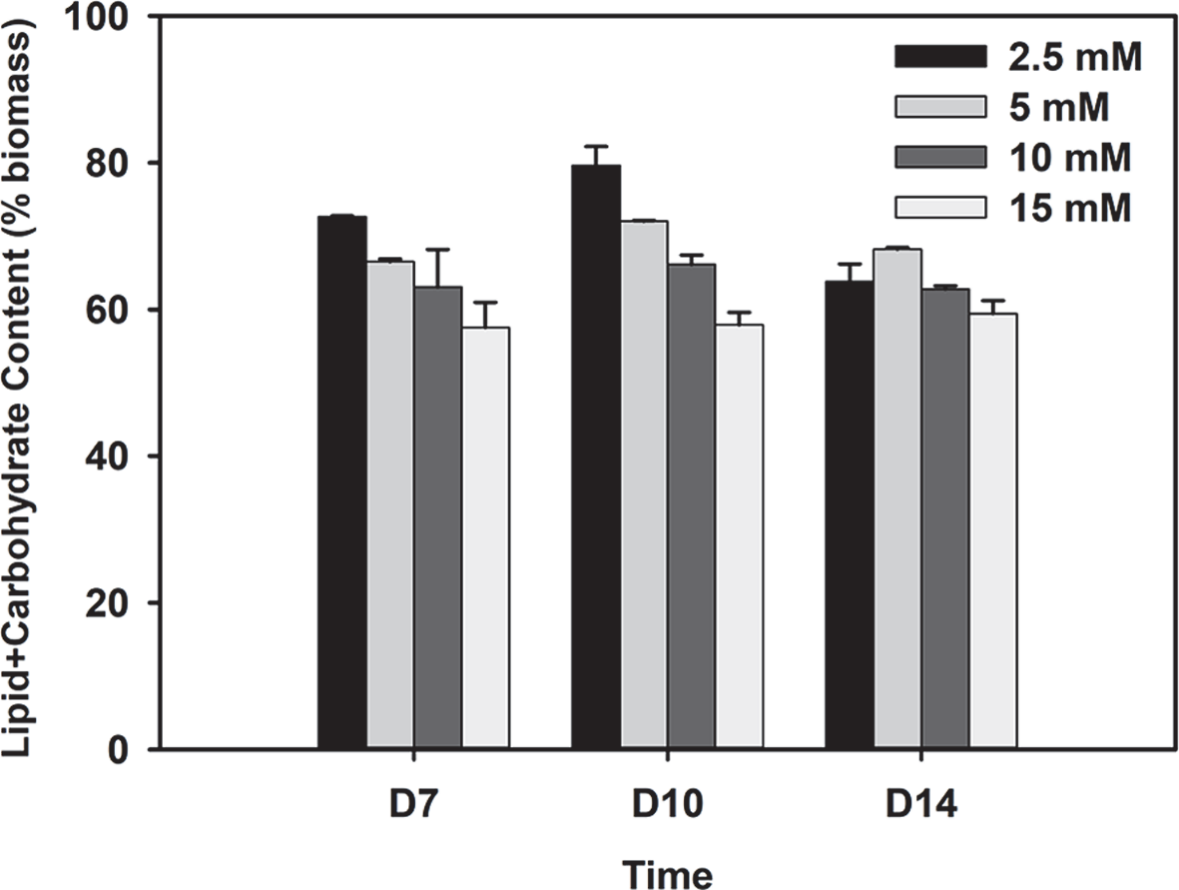
Sum of lipid and carbohydrate composition of cells grown under 2.5 to 15 mM nitrate concentrations.

**Table 1:** Elemental composition of *Chlorella* sp. ABC-001 grown under various nitrate concentrations.

Nitrate conc. (mM)	Carbon content (wt.%)		Nitrogen content (wt.%)	

	Day 7	Day 14	Day 7	Day 14
2.5	54.1 ± 0.1	57.6 ± 0.5	2.05 ± 0.1	1.85 ± 0.1
5	49.5 ± 0.1	54.5 ± 0.4	2.62 ± 0.1	2.34 ± 0.1
10	45.8 ± 0.5	51.9 ± 0.2	4.32 ± 0.1	3.37 ± 0.1
15	44.1 ± 0.1	49.6 ± 0.2	4.97 ± 0.1	4.30 ± 0.1

**Table 2:** Average biomass productivity and CO_2_ biofixation rate of *Chlorella *sp. ABC-001 grown under various nitrate concentrations.

Nitrate conc. (mM)	Average biomass productivity (g/l/d)		CO_2_ biofixation rate (g/l/d)	

	Day 7	Day 14	Day 7	Day 14
2.5	0.256 ± 0.004	0.196 ± 0.002	0.509 ± 0.009	0.415 ± 0.009
5	0.341 ± 0.014	0.237 ± 0.001	0.619 ± 0.024	0.473 ± 0.003
10	0.398 ± 0.008	0.306 ± 0.008	0.670 ± 0.007	0.584 ± 0.014
15	0.422 ± 0.008	0.341 ± 0.009	0.683 ± 0.012	0.619 ± 0.015

**Table 3:** Biomass productivity, CO_2_ biofixation rate, lipid and carbohydrate contents of various microalgae species.

Species	Biomass productivity(g/l/d)	CO_2_ biofixatio rate (g/l/d)	n Lipid content (%)	Carbohydrate content (%)	Sum of lipid and carbohydrate (%)	Reference
*Chloroccocum* sp.	0.380	0.770	20.2	19.7	39.9	[39]
*Scenedesmus vacuolatus*	0.490	0.880	28.0	20.3	48.3	[39]
*Scenedesmus obliquus* CNW-N	0.293	0.550	38.9	n/a	n/a	[40]
*Dunaliela tertiolecta*	0.300	n/a	30.0	48.9	78.9	[41]
*Botryococcus braunii* SAG-30.81	0.207	0.497	33.0	2.4	35.4	[42]
*Chlorella vulgaris* LEB-104	0.129	0.252	10.0	16.7	26.7	[42]
*Chlorella pyrenoidosa*	0.144	0.260	24.3	n/a	n/a	[43]
*Chlorella* sp. MRA-1	0.344	0.660	34.2	n/a	n/a	[24]
*Chlorella* sp. ESP-31	0.250	n/a	22.5	30.0-40.0	n/a	[23]
*Chlorella* sp. ABC-001	0.422	0.683	16.1	41.5	57.6	This study
*Chlorella* sp. ABC-001	0.256	0.509	47.4	32.2	79.6	This study
